# Characterization of *Cronobacter *recovered from dried milk and related products

**DOI:** 10.1186/1471-2180-9-24

**Published:** 2009-02-02

**Authors:** Walid M El-Sharoud, Stephen O'Brien, Carmen Negredo, Carol Iversen, Séamus Fanning, Brendan Healy

**Affiliations:** 1 Food Safety and Microbial Physiology Laboratory, Dairy Department, Faculty of Agriculture, Mansoura University, Mansoura, Egypt; 2 Centres for Food Safety & Food-borne Zoonomics, UCD Veterinary Sciences Centre, School of Agriculture, Food Science and Veterinary Medicine, University College Dublin, Belfield, Dublin 4, Ireland

## Abstract

**Background:**

*Cronobacter *is a recently proposed genus consisting of six genomospecies that encompass the organisms previously identified as *Enterobacter sakazakii. Cronobacter *are opportunistic pathogens and are known to cause serious infections in infants, particularly neonates. High case fatality rates have been associated with infections and acute sequelae can occur in survivors with severe ramifications on neurological development. Infant formula has been identified as one route of transmission for infection in infants. However, the primary reservoirs for subsequent contamination of foods with *Cronobacter *remain undefined due to the ubiquitous nature of these organisms. More recently, infections in adults have been reported, especially amongst the elderly and patients who are immunocompromised. To help prevent the transmission of infection, it is important to identify the main food sources for *Cronobacter*. The aim of this study was to identify and characterize *Cronobacter *isolated from dried-milk and related products available in an Egyptian food market.

**Results:**

In total sixteen *Cronobacter *strains were isolated from 152 dairy-based products. These were identified and characterized using pheno- and genotyping experiments. Real-time PCR confirmed the detection of *Cronobacter*. Following antibiotic susceptibility tests, 3 strains showed resistance to trimethoprim and/or neomycin. Phenotype profiles were generated based on key biochemical distinguishing tests. Pulsed-field gel electrophoresis (PFGE) identified 8 PFGE types amongst the collection of strains. Repetitive sequence based PCR (rep-PCR) analysis identified 3 rep-PCR types amongst the collection of strains. Sequencing of the *recN *gene was used to differentiate among the recently described species of *Cronobacter*.

**Conclusion:**

This study identified the presence of *Cronobacter *in dried milk and related products sourced from the Nile-Delta region of Egypt. Although the majority of the strains were susceptible to the antibiotics tested, resistance was observed in three isolates, highlighting the risks associated with *Cronobacter *contamination in foods. Phenotype and genotype analysis should be applied to further characterize *Cronobacter *spp. and prevent its transmission into food products.

## Background

Recent taxonomic work by Iversen *et al*. [1,2] has led to an alternative classification of the organism, *Enterobacter sakazakii*, and the proposal of a newly defined genus, *Cronobacter. Cronobacter *spp. are considered emerging opportunistic pathogens and are associated with outbreaks of infections amongst infants, in particular neonates [3-5]. Symptoms include bacteremia, necrotizing enterocolitis and meningitis, with case fatality rates as high as 80% being reported. The prognosis for survivors is also poor, with neurological development being severely affected in many cases [6]. More recently the association of *Cronobacter *with infections in adults has been investigated. Gosney *et al*. [7] described the isolation of *Cronobacter *from seven adult stroke patients. See *et al*. [8] reported a case of bacteremia in a 75 year old woman who presented with a splenic abscess. In total, thirteen cases of *Cronobacter *infections in adults have been documented from 1985 to present.

The primary origins of *Cronobacter *spp. remain unknown. Due to its ubiquitous nature, *Cronobacter *can be isolated from a wide variety of foods including milk, cheese, dried foods, meats, water, vegetables, rice, bread, tea, herbs and spices [9-14]. Surveillance studies have detected *Cronobacter *in infant formula production, food processing, households and clinical environments. Powdered infant formula (PIF) has been epidemiologically linked to cases of infection in infants, thus research has specifically focused on the monitoring of PIF products for the presence of *Cronobacter*. However, less is known regarding the prevalence of *Cronobacter *in other dairy foods. Recently, El-Sharoud *et al*. [15] examined dairy products from an Egyptian market for the occurrence of the organism. *Cronobacter *was isolated from skimmed milk and a related imitation soft cheese.

Identifying foods that may contain *Cronobacter *is important to discover the possible routes for transmission of infection. With the indication that *Cronobacter *spp. infect both infants and vulnerable adults it is important that a wider variety of foods now be evaluated. The aim of this study was firstly, to determine if *Cronobacter *could be found present in dried milk and related products and secondly, to characterize any isolates recovered.

## Methods

### Bacterial Cultures

A summary of the isolates characterized in this study can be seen in Table 1.

**Table 1:** *Cronobacter *isolated from various sources used in this study.

Isolate	Source
CFS-FSMP 1500	Fresh domiati cheese

CFS-FSMP 1501	Dried skimmed milk

CFS-FSMP 1502	Sahlab

CFS-FSMP 1503	Sahlab

CFS-FSMP 1504	Sahlab

CFS-FSMP 1505	Sahlab

CFS-FSMP 1506	Powdered infant formula

CFS-FSMP 1507	Powdered infant formula

CFS-FSMP 1508	Fresh domiati cheese

CFS-FSMP 1509	Fresh domiati cheese

CFS-FSMP 1510	Fresh domiati cheese

CFS-FSMP 1511	Environmental, milk factory

CFS-FSMP 1512	Dried skimmed milk

CFS-FSMP 1513	Dried skimmed milk

CFS-FSMP 1514	Dried skimmed milk

CFS-FSMP 1515	Dried skimmed milk

### Isolation & Identification

In total 152 dairy-based products obtained within the Nile-Delta region of Egypt were tested for the presence of *Cronobacter*. Additionally, strain CFS-FSMP 1511 from the environment of a milk powder factory was obtained from Nestlé Research Centre, Lausanne, Switzerland. Samples included full-fat milk powder (n = 15), skimmed milk powder (n = 37), dried whey (n = 5), dried ice-cream (n = 5), dried artificial cream (n = 5), Sahlab (n = 10), PIF (n = 35), stored- cheese (n = 10), and fresh Domiatti cheese (n = 10), Kariesh cheese (n = 10) and Ras cheese (n = 10) (Table 2). Collected samples represented different, commercially available brands of each product type. Domiati cheese is a traditional Egyptian, highly salted, enzyme-coagulated, soft cheese. Similarly, Ras cheese, also typically Egyptian is a hard cheese that is prepared from raw cow's milk or a mixture of cow and buffalo's milk. Kariesh cheese is a traditional Egyptian soft cheese that is produced by acid coagulation of skim milk by culturing with lactic acid bacteria. Sahlab is a dried blend consisting of dried skim milk, starch and nuts that is reconstituted in boiling water and served as a hot drink. Isolation was performed according to a modification of the International Organization for Standards Technical Specification on the detection of *E. sakazakii *(ISO/TS 22964). In brief, samples were diluted 1:10 (w/v) in buffered peptone water (BPW) (Oxoid CM0509, Hampshire UK) and homogenized. With regard to dried milk products and powders, 10 g of product was added to 100 ml BPW. For the various cheese samples, 25 g of product was added to 225 ml BPW. Following an overnight incubation at 37°C, 10 ml of the pre-enrichment culture was inoculated into 90 ml of *Enterobacteriacae *Enrichment (EE) broth and incubated overnight at 37°C. A 10 μl volume of the selective enrichment was then streaked onto a chromogenic media, DFI agar (Oxoid CM1055, Hampshire, UK).

**Table 2:** Occurrence of *Cronobacter *in dried milk and related products.

Sample	No. Samples	No. Positive Samples
Full-fat milk powder	15	0
Skimmed milk powder	37	5
Dried whey	5	0
Dried ice-cream	5	0
Dried artificial cream	5	0
Sahlab	10	4
Infant milk formulas	35	2
Environmental, Milk Factory	1	1
Stored Domiatti cheese	10	0
Fresh Domiatti cheese	10	4
Ras cheese	10	0
Kariesh cheese	10	0

**Total**	**152**	**16**

Presumptive positive isolates producing blue-green colonies were identified using Rapid ID 32E test galleries (bioMérieux Ref: 32700, France) as per the manufacturer's instructions. Isolates identified as *Cronobacter *(*E. sakazakii*) were confirmed using a modified version of the real-time PCR method described by Seo and Brackett [16]. In short, a primer set and probe targeting the *dnaG *gene located internally to the macromolecular synthesis (MMS) operon was applied [17].

The *Cronobacter *genus currently consists of six genomospecies [18]. To this end, isolates confirmed as *Cronobacter *were speciated using biochemical differentiation tests as described by Iversen *et al*. [19] and *recN *gene sequence analysis (Kuhnert P., Korczak B.M., Stephan R., Joosten H., Iversen C: Phylogeny and whole genome DNA-DNA similarity of *Enterobacter *and related taxa by multilocus sequence analysis (MLSA)).

### Antibiotic Susceptibility Testing

*Cronobacter *isolates were tested for their susceptibility to ampicillin (10 μg), compound sulphonamides (300 μg), furazolidone (15 μg), gentamicin (10 μg), neomycin (30 μg), spectinomycin (100 μg), streptomycin (10 μg), and trimethoprim (5 μg) using the Kirby-Bauer disc diffusion method [20]. Antibiotic disks were obtained from Oxoid, Hampshire, UK.

### Molecular Subtyping

Pulsed-field gel electrophoresis (PFGE) was applied as described previously [21]. Analysis was carried out using BioNumerics software V3.0 (Applied Maths, Sint-Martens-Latem, Belgium). A dendrogram was generated using the DICE coefficient and unweighted pair group method with arithmetic mean (UPGMA). A band tolerance and optimization coefficient of 1.5% was applied.

Repetitive sequence-based (rep-PCR) amplification was performed using an automated rep-PCR system as previously described [22]. Analysis was performed using Diversilab^® ^software V3.3 (Diversilab^®^, bioMérieux, France). Isolate similarity was calculated using the Pearson Correlation (PC) coefficient.

### *recN *Gene Sequencing

*recN *gene sequencing was performed by Fasteris SA (Plan-les-Ouates, Switzerland) using a modified version of the method described by Kuhnert *et al*. (Kuhnert P., Korczak B.M., Stephan R., Joosten H., Iversen C: Phylogeny and whole genome DNA-DNA similarity of *Enterobacter *and related taxa by multilocus sequence analysis (MLSA)). PCR reactions were carried out in 3 × 15 μl volume, which were then pooled together. The thermo cycling conditions employed were as follows: 95°C for 3 min, followed by 30 cycles comprising 95°C for 30 s, 54°C for 30 s and 72°C for 2 min. A final extension of 72°C for 5 min was applied. The primers used for *recN *sequencing were:

**Es-L_seq1**   5'-CTGGCACAATTAACCATCAGTAA-3'

**Es-L_seq2**   5'-CTGGCACAATTAACCATCAGCAA-3'

**Es-R_seq**   5'-TGGGTAACGCACATCACCTGAGT-3'

**Figure 1:**
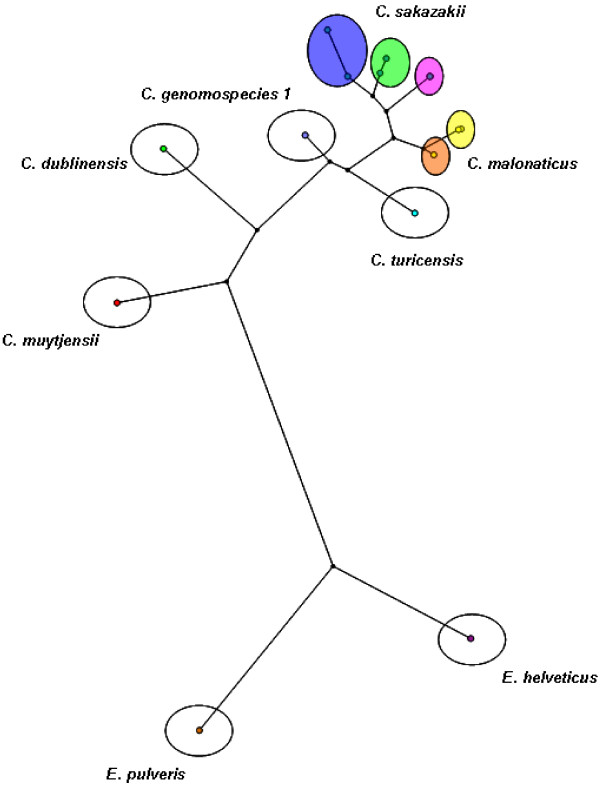
***recN *gene sequencing clustering analysis of *Cronobacter *species (Colours relate to the phenotypes in Table 3)**.

A maximum parsimony phylogenetic tree (Figure 1) was generated using BioNumerics software (Applied Maths, Belgium). Gaps were not considered an extra state. The Jukes-Cantor correction was used to compensate for divergence being a logarithmic function of time due to the increased probability of a second substitution at a nucleotide site slowing the increase in the count of differences as divergence time increases [23]. Felsenstein bootstraps (1,000 simulations) were applied to assess the level of confidence for each clade of the observed trees based on the proportion of bootstrap trees showing the same clade [24]. The topology of the maximum parsimony tree was optimized using simulated annealing. [This is a heuristic approach that occasionally accepts a worse tree during the course of the search allowing it to escape local optima. This method is more economical than the more usual heuristic searches (stepwise addition and hill-climbing), which can require many random re-starts, especially with large data matrices].

## Results

### Isolation & Identification

A total of sixteen *Cronobacter *strains were isolated from various food products (Table 1). Some of the non-*Cronobacter *strains isolated included *Citrobacter freundii, Enterobacter cloacae, Proteus vulgaris *and putative *Vibrio cholerae*. Presumptive positive isolates produced blue-green colonies on DFI agar and were identified as *Cronobacter *(*E. sakazakii*) using ID 32E test strips. Real-time PCR analysis confirmed the detection of *Cronobacter *isolates. Biochemical tests were performed in order to distinguish the phenotypes of the *Cronobacter *isolates and contribute to the speciation of the collection of strains. The results of these tests are shown in Table 3.

**Table 3:** Results of pheno- and genotyping of *Cronobacter *isolates.

Isolate	Species	AMG	DUL	IND	INO	MAL	rep-PCR	PFGE
CFS-FSMP 1504	*C. sakazakii*	**+**	**-**	**-**	**+**	**-**	B	7
CFS-FSMP 1505	*C. sakazakii*	**+**	**-**	**-**	**+**	**-**	B	7
CFS-FSMP 1502	*C. sakazakii*	**+**	**-**	**-**	**+**	**-**	B	8
CFS-FSMP 1503	*C. sakazakii*	**+**	**-**	**-**	**+**	**-**	B	8
CFS-FSMP 1506	*C. sakazakii*	**+**	**-**	**-**	**+**	**-**	B	8
CFS-FSMP 1511	*C. sakazakii*	**+**	**-**	**-**	**+**	**-**	C	2
CFS-FSMP 1512	*C. sakazakii*	**+**	**-**	**-**	**+**	**-**	C	2
CFS-FSMP 1515	*C. sakazakii*	**+**	**-**	**-**	**+**	**-**	C	2

								

CFS-FSMP 1513	*C. sakazakii*	**+**	**-**	**-**	**+**	**+**	C	1
CFS-FSMP 1514	*C. sakazakii*	**+**	**-**	**-**	**+**	**+**	C	1
CFS-FSMP 1501	*C. sakazakii*	**+**	**-**	**-**	**+**	**+**	C	3

								

CFS-FSMP 1507	*C. sakazakii*	**-**	**+**	**+**	**-**	**-**	B	6

								

CFS-FSMP 1500	*C. malonaticus*	**+**	**-**	**-**	**-**	**+**	A	4
CFS-FSMP 1508	*C. malonaticus*	**+**	**-**	**-**	**-**	**+**	A	4
CFS-FSMP 1510	*C. malonaticus*	**+**	**-**	**-**	**-**	**+**	A	4

								

CFS-FSMP 1509	*C. malonaticus*	**+**	**-**	**-**	**-**	**-**	A	5

**AMG **1-0-methyl-alpha-D-glucopyranoside, **DUL **dulcitol, **IND **indole, **INO ***myo*-inositol, **MAL **Malonate;

Bold are indicative of different phenotype profiles

### Antibiotic Susceptibility

The antibiotic susceptibility testing indicated that all isolates were susceptible to streptomycin, compound sulphonamides, ampicillin, gentamicin, spectinomycin and furazolidone. Isolates CFS-FSMP 1500 and CFS-FSMP 1512 were found to be resistant to neomycin and isolate CFS-FSMP 1510 was reported as resistant to trimethoprim and neomycin. All other isolates were found susceptible to these two antimicrobial agents (Table 4).

**Table 4:** Results of antimicrobial susceptibility testing of *Cronobacter *isolates.

Isolate	S	S3	AMP	W	CN	SH	FR	N
CFS-FSMP 1500	15.70	18.30	19.94	23.78	19.20	16.99	19.60	6.29*

CFS-FSMP 1501	17.56	28.72	25.21	29.26	21.47	22.16	21.83	17.97

CFS-FSMP 1502	16.54	28.72	20.30	22.98	21.28	22.37	21.30	17.75

CFS-FSMP 1503	18.67	24.94	23.36	25.80	23.17	22.53	23.14	18.95

CFS-FSMP 1504	17.86	30.42	21.97	24.31	22.12	23.05	22.68	17.92

CFS-FSMP 1505	18.33	29.49	22.40	26.27	21.79	24.27	22.73	19.03

CFS-FSMP 1506	18.74	31.27	22.24	25.45	23.09	23.27	23.36	19.31

CFS-FSMP 1507	17.91	30.37	22.80	25.38	21.71	28.50	23.30	18.88

CFS-FSMP 1508	17.95	32.25	22.89	27.49	20.81	21.05	23.21	17.85

CFS-FSMP 1509	18.27	23.43	22.74	26.38	21.55	22.36	22.55	17.89

CFS-FSMP 1510	17.51	26.33	22.95	7.02*	22.10	23.20	22.93	6.46*

CFS-FSMP 1511	18.37	30.95	24.75	26.40	22.30	23.23	22.46	18.53

CFS-FSMP 1512	18.53	30.55	24.78	26.90	22.63	19.83	23.41	11.95*

CFS-FSMP 1513	16.16	31.73	25.49	26.08	20.95	20.62	22.87	18.58

CFS-FSMP 1514	17.45	25.54	24.14	25.75	22.73	23.28	23.30	18.27

CFS-FSMP 1515	16.11	30.74	24.79	24.66	21.21	22.09	20.76	17.51

**S **streptomycin, **S3 **compound sulphonamides, **AMP **ampicillin, **W **trimethoprim, **CN **gentamicin, **SH **spectinomycin, **FR **furazolidone, **N **neomycin; **Green **= susceptible, *Denotes resistance; diameter of inhibition zone was measured in mm.

### PFGE Analysis

**Figure 2:**
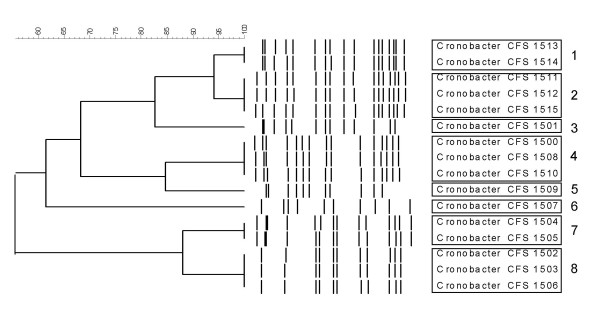
**PFGE analysis showing the clustering of *Cronobacter *isolates recovered from dairy products**.

Macrorestriction of *Cronobacter *genomic DNA with XbaI yielded 10 to 17 DNA fragments ranging in size from 48.5 to 1,000 kbp. A dendrogram was compiled which illustrates the fingerprint pattern similarities between the various *Cronobacter *isolates (Figure 2). In total, 8 pulse-types (denoted 1 through 8) were identified that showed indistinguishable similarity.

### rep-PCR Analysis

**Figure 3:**
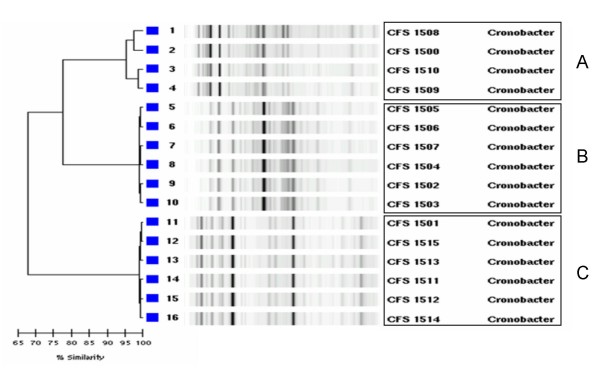
**rep-PCR analysis illustrating the relatedness of *Cronobacter *isolates recovered from dairy products**.

The rep-PCR typing yielded between 18 and 25 DNA fragments that ranged in size from 150 to 3,500 bp. A dendrogram representing the genetic relatedness amongst the isolates was composed (Figure 3). Amongst the collection, 3 rep-PCR cluster groups (denoted A, B and C) were identified that exhibited identical similarity.

### *recN *Gene Sequencing

The results of the *recN *sequence analysis determined that two *Cronobacter *species, *C. sakazakii *and *C. malonaticus*, had been isolated in this study. The sequences of the *recN *gene analysis for the following strains were deposited in the GenBank database: CFS-FSMP 1501 (FJ624476), CFS-FSMP 1502 (FJ624470), CFS-FSMP 1504 (FJ624469), CFS-FSMP 1507 (FJ624473), CFS-FSMP 1509 (FJ624471), CFS-FSMP 1510 (FJ624472), CFS-FSMP 1513 (FJ624474) and CFS-FSMP 1515 (FJ624475). A maximum parsimony tree was produced that displays the genetic relationship amongst the collection of strains (Figure 1). For comparison purposes a representative for each of the other *Cronobacter *spp., *C. dublinensis *(EU569474), *C. genomospecies *1 (EU569479), *C. muytjensii *(EU569492), *C. turicensis *(EU569523) and two novel *Enterobacter *species, *E. helveticus *(EU569447) and *E. pulveris *(EU569451), which represent the closest related species of *Cronobacter*, were also included in the analysis.

## Discussion

The focus of this study was to test a collection of dried milk and related products available in Egypt for the presence of *Cronobacter*. While PIF has been identified as one vehicle of transmission for infection in infants, less is known regarding other dried dairy products. More recent reports have also identified *Cronobacter *infections in immunocompromised adults, further highlighting the need to identify these organisms' primary origin for contamination. The food products tested included milk powders, PIF, dried whey, dried ice-cream, Sahlab and cheese and all were obtained from the Nile-Delta region of Egypt. In total, a collection of sixteen *Cronobacter *isolates were recovered from the foods tests and these were characterized using both pheno- and genotyping methods. The results of the biochemical assays identified the presence of 5 phenotype profiles amongst the collection of isolates (Table 3).

PFGE and rep-PCR analysis was performed for molecular characterization of the isolates. PFGE typing identified 8 pulse-type cluster groups exhibiting ≥ 95% similarity. Analysis using rep-PCR typing identified 3 cluster groups that showed ≥ 95% similarity. Interestingly, rep-PCR clustered all the *C. malonaticus *isolates into a single cluster, denoted as rep-PCR type A, while the *C. sakazakii *isolates formed two distinct clusters, rep-PCR types B and C. Isolates CFS-FSMP 1507 and 1509 produced unique phenotype profiles when compared with the other strains in the collection. PFGE analysis also grouped the latter two isolates into distinct clusters, pulse-types 6 and 5 respectively. Further work is needed to determine whether or not these strains represent unique subtypes of *C. sakazakii*. Sequencing of the *recN *gene was applied to further characterize the isolates and confirm the species identification. This method was chosen as it has shown a higher discriminatory power with regard to the speciation of *Cronobacter *isolates when compared to 16S rRNA sequencing (Kuhnert P., Korczak B.M., Stephan R., Joosten H., Iversen C: Phylogeny and whole genome DNA-DNA similarity of *Enterobacter *and related taxa by multilocus sequence analysis (MLSA)). The method identified two *Cronobacter *species recovered in this study, *C. sakazakii *and *C. malonaticus*. Figure 1 outlines the genetic relationship between the *Cronobacter *species type strains, the isolates from this study and the genetically closest *Enterobacter *species. As expected, the isolates recovered from the foods studied, clustered with the type strains of *C. sakazakii *and *C. malonaticus*.

Antimicrobial susceptibility testing indicated that all isolates were susceptible to ampicillin, compound sulphonamides, furazolidone, gentamicin, spectinomycin and streptomycin. These findings are in agreement with the data obtained by Stock and Wiedemann [25]. In their study they identified *Cronobacter *as being more susceptible to β-lactam antibiotics, including ampicillin, when compared with the *Enterobacter *species, *E. amnigenus, E. cancerogenus *and *E. gergoviae*. Interestingly, the *Cronobacter *isolates screened in their study were naturally susceptible to neomycin. The isolates CFS-FSMP 1500, 1510 and 1512 were resistant to this antibiotic. Neomycin is an aminoglycoside antibiotic, the mode of action of which is to bind to the 30S ribosomal subunit of bacteria. A possible reason behind this observed resistance could be an alteration to the binding site protein of the 30S subunit. Such an occurrence has previously led to streptomycin resistance, another aminoglycoside compound. In the Stock and Wiedemann study [25] all *Cronobacter *and *Enterobacter *strains tested were susceptible to antifolate compounds. However, in our study isolate CFS-FSMP 1510 was resistant trimethoprim. Trimethoprim is an antifolate compound and acts by inhibiting dihydrofolate reductase enzymes in susceptible bacteria. Resistance in Gram-negative bacteria has previously been reported and it is believed that the mechanism of resistance lies within the expression of plasmid and/or transposon mediated dihydrofolate reductase genes.

## Conclusion

This study identified and characterized *Cronobacter *isolates recovered from dried milk and related food products. Although the majority of the strains were susceptible to the panel of antibiotics tested, resistance patterns observed in three isolates may indicate increasing risks to public health associated with the presence of *Cronobacter *in foods. Phenotypic and genotypic analysis should be applied to further monitor and characterize the presence of *Cronobacter *in food production environments and prevent its transmission thereby improving food safety and quality.

## Authors' contributions

WME isolated the cultures and contributed to the outline of the study. SOB performed PFGE analysis of the isolates and contributed to the drafting of the manuscript. CN performed the biochemical profiling of the collection of strains and participated in drafting the manuscript. CI carried out recN gene sequence analysis and alignments and helped draft the manuscript. SF conceived of the study, and participated in its design and helped to draft the manuscript. BH coordinated the study and carried out real-time PCR detection, rep-PCR molecular subtyping of the isolates and drafted the manuscript. All authors read and approved the final manuscript.
